# Winter Clothing

**Published:** 1886-02

**Authors:** 


### WINTER CLOTHING.

Nearly all the people of this country, or, at least, more than one half of them, are affected with cold, and most likely at the commencement of the cold season. If we are allowed to examine the majority of cases we would find that by far the largest number were by persons who had neglected to change their clothing at the begining of cold weather and many others who boast. "O I never wear underclothes:" How often we have heard this silly boast. Persons who know the nature of woolen and cotton, will never make a boast of this kind; more than this, they should always wear woolen next the skin in summer, whether they live in a warm climate or a cold one. It is not merely the warmth of the goods, but it has a more decided effect, specially in warm weather. It is capable, according to its texture and in virtue of its composition, of better adaptation, in respect of temperature, to the needs of various climates and the changes of seasons than any other dress material. Moreover, it exhibits a special faculty for absorbing and distributing moisture. It is this property especially, which renders it the natural next covering of the constantly perspiring skin. If one be engaged, for example, in active exercise of limb, a linen fabric will absorb what products of transudation it can till it is wet, but will leave much moisture unabsorbed upon the clammy surface; whereas a flannel, from its more spongy nature, will rest upon a skin which it has nearly dried, and be but damp itself. It is obvious, then, that in the event of an after-chill—and this occurs in Summer as in Winter—the body is, in the latter case, most favorably disposed to resist it. Fannel is not less cleanly than linen, though it may appear less white; and if the wearer bathe daily, it is surprising how long it will retain its purity. The skin irritation to which it sometimes gives rise, is usually associated with coarseness of quality or freshnes of manufacture, and is, with nearly all who have experienced it, a merely transient conditon. Women, as well as men, we repeat, but above all, children and the aged, who are alike particularly apt to take cold, should certainly adopt a woolen material for their customary undergarments. It is easily possible to adjust the texture to the season, so that it shall be warm enough in Winter, and not too warm in Summer. The hard-working men in our furnaces, and machine works, you will find, in the hottest days in summer, although where there is a temendous heat from the fires, always wear flannel, as well to protect them from the sudden change of atmosphere from the sparks of coal and hot iron that are constantly moving about.

